# Delineation of single-cell Altas provides new insights for development of coronary artery lesions in Kawasaki disease: bad and good signaling molecules

**DOI:** 10.3389/fped.2025.1596643

**Published:** 2025-06-25

**Authors:** Qiuping Lin, Xin Lv, Qingzhu Qiu, Lianni Mei, Liqin Chen, Sirui Song, Wei Liu, Xunwei Jiang, Min Huang, Libing Shen, Tingting Xiao, Lijian Xie

**Affiliations:** ^1^ Department of Cardiology, Shanghai Children’s Hospital, School of Medicine, Shanghai Jiao Tong University, Shanghai, China; ^2^ Department of Pediatrics, JinShan Hospital, Fudan University, Shanghai, China; ^3^ Institute of Pediatric Infection, Immunity, and Critical Care Medicine, School of Medicine, Shanghai Jiao Tong University, Shanghai, China; ^4^ Longhua Hospital, Shanghai University of Traditional Chinese Medicine, Shanghai, China

**Keywords:** Kawasaki disease, coronary artery lesion, CD14 monocyte, signaling molecule, MCH-II, resistin

## Abstract

**Background:**

Kawasaki Disease (KD) is a vasculitis syndrome featured with a high and persistent fever in children. It is the leading cause of coronary artery lesions (CALs) for children in developed countries.

**Methods:**

Single-cell RNA sequencing analyses were performed for the peripheral blood mononuclear cells from three KD non-CAL patients before/after IVIG treatment (KD BT and KD AT), three KD CAL patients before IVIG treatment (CAL BT), and three KD CAL patients after IVIG treatment (CAL AT).

**Results:**

Overall expression analyses show immunoglobulin and adaptive immunity related genes are commonly upregulated in CAL BT and AT patients while antimicrobial and innate immunity related genes are commonly downregulated in them. Pseudo-time analyses demonstrate that CAL BT patients have a disorganized cell development trajectory with multiple overlapped cell linages and CAL AT patients have a dysregulated B cell developmental trajectory featured with a mixed monocyte and B lineage. In gene branch pseudo-time analyses, the repressed expression of SPI1 and MT2A are found in CAL BT patients, which is similar to their expression patterns in KD BT patients; while the early elevated expression of SPI1 and MT2A could partly explain the dysregulated B cell development in CAL AT patients. Cell communication analyses demonstrates that CAL BT patients have the lower number of inferred cell-to-cell interactions and the weakest interaction strength among four groups, whereas CD14 monocytes in CAL AT and KD BT patients have strong cell-to-cell interaction strength which may contribute to CAL or KD pathogenesis. In the monocytes of CAL patients, MCH-II is a significantly increased signal and RESISTIN is a significantly decreased signal compared to non-CAL counterpart.

**Conclusions:**

Our results suggest that MCH-II is a bad signal for indicating CAL development while RESISTIN is a good signal for protecting from CAL development.

## 1 Introduction

Kawasaki disease (KD) is a form of small and medium-sized vasculitis featured with high fever and its etiology is unknown until now. KD usually occurs in the children under age of 5 and causes coronary artery dilatation or aneurysm. It is the most common cause of acquired heart disease in children in developed countries (1). If KD was left untreated, the incidence of coronary artery lesions (CALs) ranges from approximately 20% to 25% (2). Intravenous immunoglobulin (IVIG) is the standard treatment for KD, which could reduce the risk of CALs to 5% (3).

The pathological process of CALs is not clear at present. Studies pointed out that coronary vasculitis begins 6–8 days after the onset of KD and then the inflammation rapidly invades all layers of the artery (4). Coronary artery aneurysms (CAAs) have been reported to regress in size after 4–8 weeks in an acute episode, but it may take several years for coronary artery diameters to return to normal (1). Orenstein et al. identified three interrelated pathologic processes in KD with CALs, including necrotizing arteritis, subacute chronic arteritis, and luminal myofibroblast proliferation (5).The endothelium, located in the inner surface of coronary arteries, serves as the interface between the circulation and the vascular media or adventitia, so endothelial dysfunction (ED) is believed to contribute to the development of CALs (6, 7). Studies suggest that CALs formation in KD are related to various genetic factors, such as HLA-E, HLA-B, CASP3, ITPKC, and etc. (8–10). Inflammatory cell activation, pro-inflammatory cytokines production, the innate immune system and the adaptive immune system are all involved in vasculitis formation (11). The mechanism of CALs formation still remains unknown, which brings challenges to the prevention and treatment of CALs.

Unlike traditional bulk RNA sequencing methods, single-cell RNA sequencing (scRNA-Seq) allows the investigation of individual cells. This technology reveals distinct transcriptional cell states and facilitates the identification of rare cell populations (12). Based on the analytical power of scRNA-Seq, the exploration of cell-to-cell communication has gained significant attention (13). By analyzing cell communication patterns to infer and visualize signaling pathways between cells (14), cell-to-cell communication analysis can elucidate how different cell types interact in a dynamic microenvironment. This understanding of cell communication atlas is crucial for deciphering complex biological processes, such as immune responses and tissue homeostasis (15). scRNA-Seq combined with cell communication analysis were applied for pancreatic, and breast cancers, elucidating complex tumor microenvironments and identifying novel therapeutic approaches (16–18). What's more, these methodologies were applied for immune-mediated diseases, delineated disease-specific ligand-receptor networks and shed light on shared immune responses across tissues and diseases (19). Immune disorder inevitably happens in KD patients. By integrating scRNA-Seq with cell-cell communication analysis, we can investigate immune cell dynamics, differentially expressed genes, and altered biochemical pathways, as well as characterize cellular interactions. This approach will provide immune-specific insights into Kawasaki disease (KD) with coronary artery lesions (CAL).

So far, few studies have explored cellular communication at the single-cell level in KD patients with CALs. Here we performed single-cell transcriptomic sequencing on six children with KD or KD CAL. Total twelve scRNA-Seq samples were acquired and six of them developed CALs before or after IVIG treatment. Cell-to-cell communication was analyzed among different sample groups. Our study aims to explore cellular panorama and communication cross talks in KD, hopefully contributing to the understanding of KD with CAL's etiology and potential therapeutic strategies.

## 2 Results

### 2.1 Clinical characteristics of the participants

We collected 9 peripheral blood samples from six KD patients. The patients were divided into 2 groups, KD patients with CALs (the CAL group) and KD patients without CALs (the KD group). For KD group, peripheral blood samples were collected on the 5th day after the onset of fever before IVIG treatment and 24 h after IVIG treatment and subsidence of fever (KD BT and KD AT). For CAL group, blood samples were only collected 24 h after IVIG therapy (CAL AT). The patients in KD group were aged 2.1–5.6 years and the mean age was 4.4 years. Two of them were diagnosed with complete KD and the other one was diagnosed with incomplete KD. The patients in CAL AT group were aged 1.0–2.0 years and the mean age was 1.5 years. All of them were diagnosed with complete KD. Two groups received high-dose IVIG standard therapy combined with aspirin treatment after diagnosis with KD. All patients responded to IVIG treatment. We collect the laboratory data about risk factors of CALs including WBC, percentage of neutrophils, Hb, PLT, CRP, ESR, D-dimer, AST, Albumin, and sodium (20–23). Comparisons of the clinical characteristics and laboratory data between KD group and CAL group were summarized in Tables 1, 2. Due to the limited samples, we did not find significant differences between the KD group and CAL AT group in the laboratory data.

**Table 1:** Clinical characteristics of participants.

Characteristic	KD1	KD2	KD3	CAL1	CAL2	CAL3
Age (years)	2.1	5.6	5.4	1.5	1.0	2.0
Sex (M/F)	M	M	F	F	F	M
Fever (days)	5	5	5	14	5	5
Conjunctivitis (yes/no)	Y	Y	Y	Y	Y	Y
Oral changes (yes/no)	Y	Y	Y	Y	Y	Y
Extremity changes (yes/no)	Y	N	N	Y	Y	Y
Rash (yes/no)	Y	Y	N	N	Y	N
Cervical lymphadenopathy (yes/no)	Y	Y	Y	Y	Y	Y
IVIG responsive (yes/no)	Y	Y	Y	Y	Y	Y
Complete KD (yes/no)	Y	Y	N	Y	Y	Y
CAL (yes/no)	N	N	N	Y	Y	Y
Zmax at the time of blood draw	1.02	1.84	0.71	4.11	4.25	2.66

**Table 2:** Laboratory data of participants before IVIG treatment.

Laboratory data	KD1	KD2	KD3	CAL1	CAL2	CAL3
WBC (10^9^/L)	8.86	17.18	24.28	10.1	14.57	25.76
NEU (%)	78.4	75.4	83.3	58.3	63.6	67.1
Hb (g/L)	111	109	121	94	102	130
PLT (10^9^/L)	262	244	462	361	373	283
ESR (mm/h)	43	110	73	37	75	43
CRP (mg/L)	18	55	61	6	71	94
D-dimer (mg/L FEU)	4.46	0.51	1.21	0.7	2.67	0.36
AST (U/L)	33	17	35	60	38	27
Albumin (g/L)	37.81	44.63	38.18	35.62	37.1	41.23
Na^+^ (mmol/L)	134	130	135	134	135	136

WBC, white blood cell; NEU, neutrophil; Hb, hemoglobin; PLT, platelet; ESR, erythrocyte sedimentation rate; CRP, C-reactive protein; AST, aspartate aminotransferase; Cr, creatinine; Na+, serum sodium.

For the KD patients with CALs before IVIG treatment (CAL BT), we used the public data from the Gene Expression Omnibus (GEO) database (GSE254657).

### 2.2 Delineation of single-cell transcription atlas of PBMCs in CAL and KD patients

**Figure 1:**
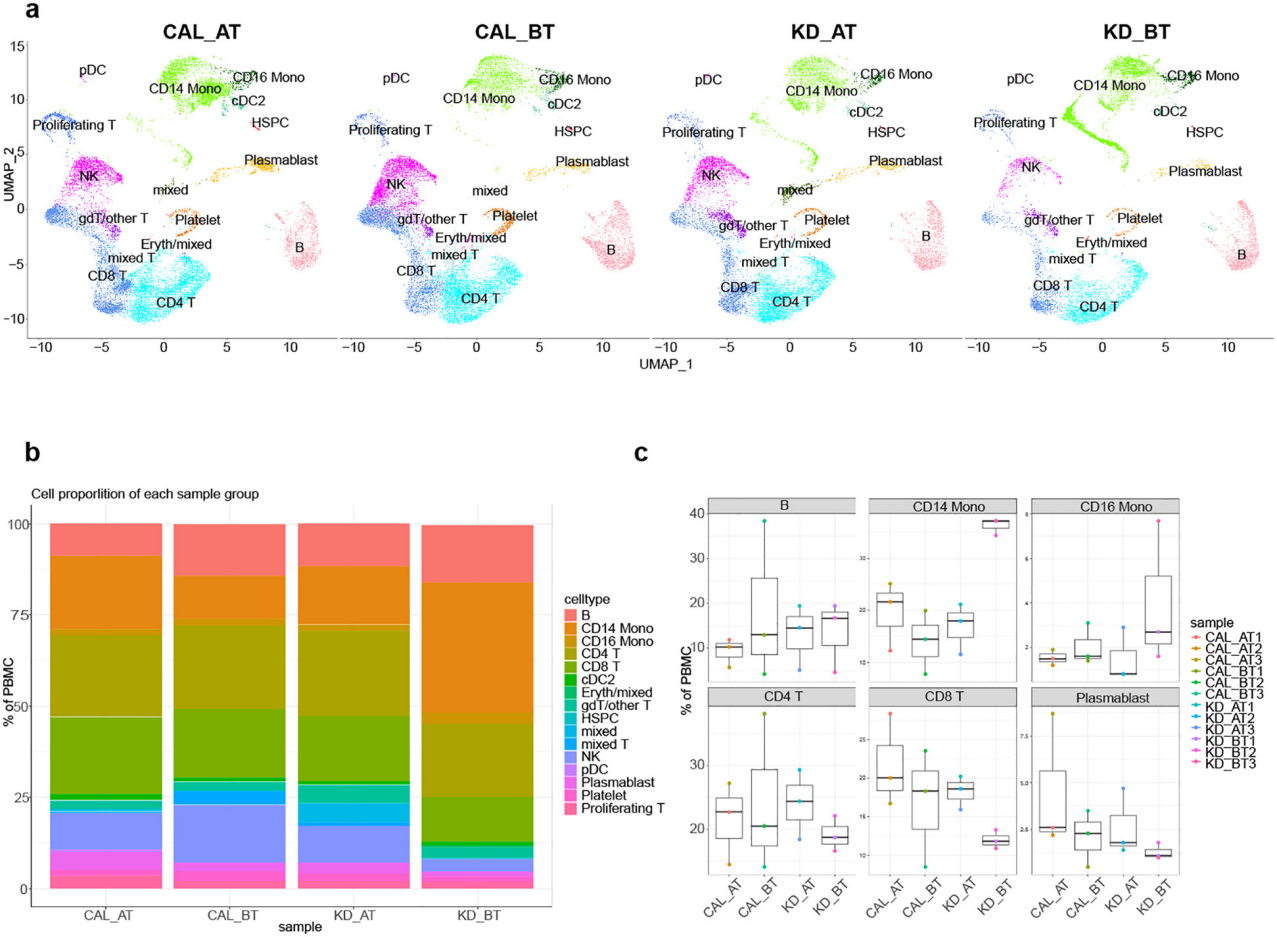
Single-cell profiling of PBMCs in CAL and KD patients. **(a)** The integration single-cell profiling analysis of twelve samples from KD CAL patients after IVIG treatment (CAL AT), KD CAL patients before IVIG treatment (CAL BT), KD non-CAL patients after IVIG treatment (KD AT), and KD non-CAL patients before IVIG treatment (KD BT). **(b)** The proportion of different cell types in each sample groups. **(c)** Comparison of the proportion of major cell types among four sample groups.

To delineate the overall single-cell transcriptome features of CAL and KD patients, we collected the PBMCs from 3 CAL AT patients and 3 KD patients. 3 published samples of CAL BT PBMCs were also added in this study. 3 CAL AT patients in this study were received IVIG treatment. The PBMCs of KD patients were collected before and after IVIG treatment. Thus, there were four sample groups in our study: CAL AT patients, CAL BT patients, KD BT patients, and KD AT patients. After quality control and filter, the total number of detected cells was 77,758, including 31,530 cells from CAL AT patients, 31,586 from CAL BT patients, 26,503 cells from KD AT patients, and 19,725 cells from KD BT patients. To describe the scRNA-Seq profiles in four PBMC groups, we first integrated 12 samples together and clustered the cells across samples according to their expression features (Figure 1A and Supplementary Figure S1a). The detected cells could be classified into 16 major cell types including B cells, CD4T cells, CD8T cells, CD14 monocytes (CD14 mono), CD16 monocytes (CD16 mono), plasmacytoid dendritic cells (pDC), type 2 conventional dendritic cells (cDC2), erythrocytes and mixed cells (Eryth/mixed), gamma-delta cells and other T cells (gdT/other T), natural killer cells (NK), plasma blast cells (Plasmablast), platelets, hematopoietic stem and progenitor cells (HSPCs), mixed proliferating T cells (proliferating T), mixed cells (mixed), and mixed T cells (mixed T). Both multimodal PBMC reference dataset and the canonical gene markers were used to validate major cell types (Supplementary Figures S1b,c).

### 2.3 The proportions of different cell types among CAL patients and KD patients

The percentages of major cell types for each patient are show in Figure 1B. Our scRNA-Seq data demonstrated that KD AT patients had an increased CD8T cells (*p* < 0.05) and a decreased percentage of CD 14 monocytes (*p* < 0.05) compared with KD BT patients, which were also well established by previous study (24).We observed no such increased and decreased percentages of major cell types between CAL AT patients and CAL BT patients (Figure 1C). Notably, KD BT patients have the highest percentage of CD 14 monocytes. It proposes that IVIG treatment is at least effective for correcting the tilted major cell percentages in KD BT patients.

### 2.4 Overall expression features of all single cells in CAL and KD patients

**Figure 2:**
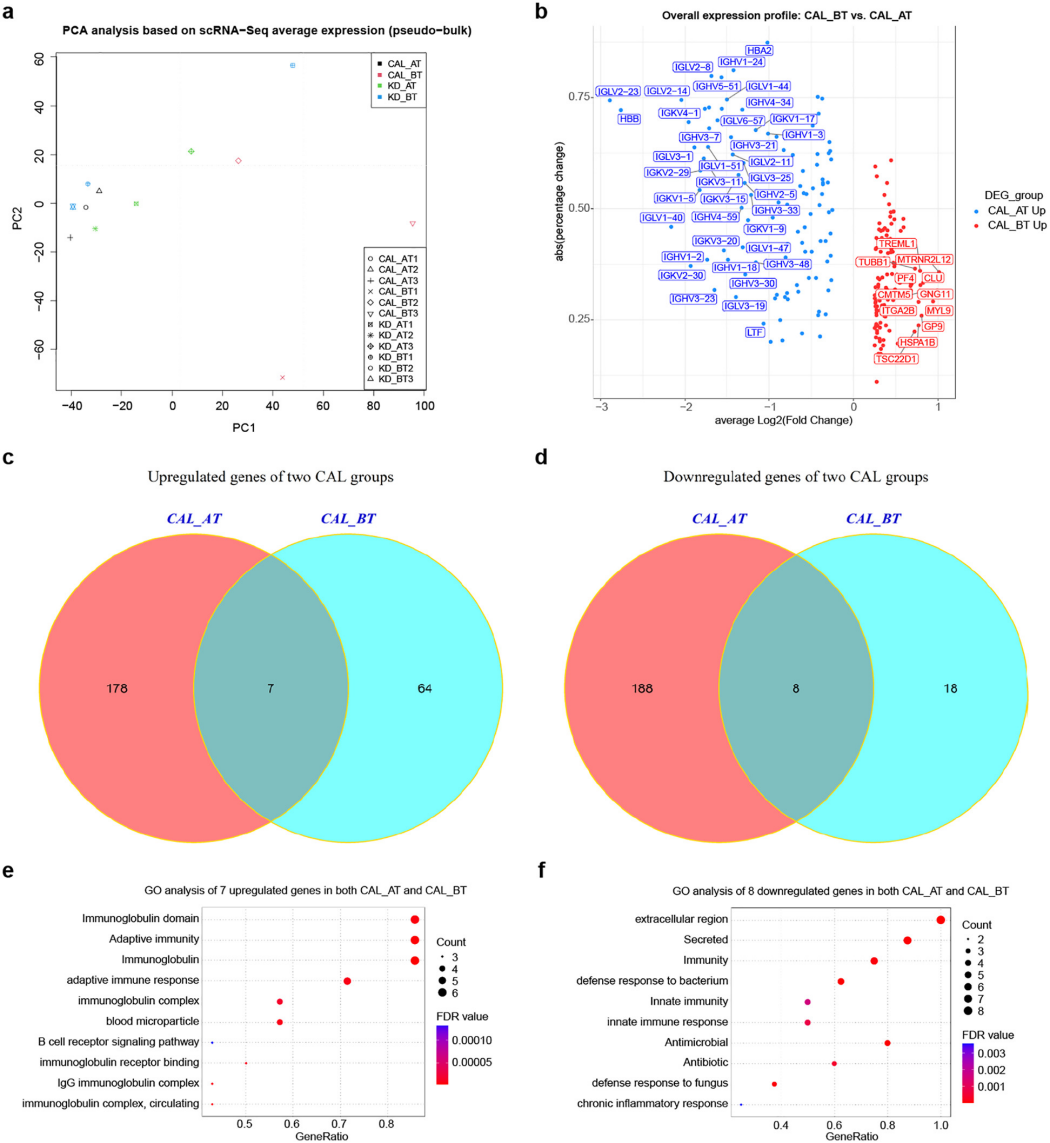
Expression analyses of all single-cells in CAL and KD patients. **(a)** Principal component analysis of twelve samples based on average cell expression values (pseudo-bulk RNA-Seq clustering). **(b)** Volcano plot of upregulated and downregulated genes between CAL AT and CAL BT patients. **(c)** Venn diagram of upregulated genes in CAL AT and KD AT comparison (labeled as CAL AT) and CAL BT and KD BT comparison (labeled as CAL BT). **(d)** Venn diagram of downregulated genes in CAL AT and KD AT comparison (labeled as CAL AT) and CAL BT and KD BT comparison (labeled as CAL BT). **(e)** GO term enrichment analysis of 7 shared upregulated genes in two CAL groups. **(f)** GO term enrichment analysis of 8 shared downregulated genes in two CAL groups.

We examined the expression features of all single cells in CAL and KD patients. First, we used the average expression value from each gene in scRNA-Seq to simulate the RNA-Seq expression profile (pseudo-bulk RNA-Seq) for 12 samples and performed PCA analysis using 5,000 most differentially expressed genes. The result shows that each sample group has a trend of gathering together, which indicates that each sample group has a similar expression background (Figure 2A). The differentially expressed genes (DEGs) were identified between CAL BT and CAL AT patients. There are 115 upregulated genes identified in CAL BT patients and 106 upregulated genes identified in CAL AT patients (Figure 2B). Immunoglobin genes are highly expressed in CAL BT patients, which is consistent with the basic pathological process before IVIG treatment. To understand the CAL development, we further searched the DEGs among two comparison groups: CAL AT patients vs. KD AT patients, and CAL BT patients vs. KD BT patients. Four sets differentially expressed genes (DEGs) were identified in CAL patients using KD patients as match groups. 185 upregulated genes detected in CAL AT vs. KD AT group and 71 upregulated genes detected in CAL BT vs. KD BT group (*p* < 0.05, Figure 2C). 196 downregulated genes detected in CAL BT vs. KD BT group and 26 downregulated genes detected in CAL BT vs. KD BT group (*p* < 0.05, Figure 2D). There are 7 shared upregulated genes in two CAL group (Figure 2C). There are 8 shared downregulated genes in two CAL groups (Figure 2D). GO analyses show that the shared upregulated genes in CAL patients are mainly involved in immunoglobulin and adaptive immunity while the shared downregulated genes in CAL patients are mainly involved in innate immunity and innate immune response (Figures 2e,f). Thus, overall expression features of all single cells in CAL patients propose that they have a strong adaptive immune response and weak innate immune response compared to KD patients.

### 2.5 Pseudo-time analyses of PBMCs in CAL and KD patients

**Figure 3:**
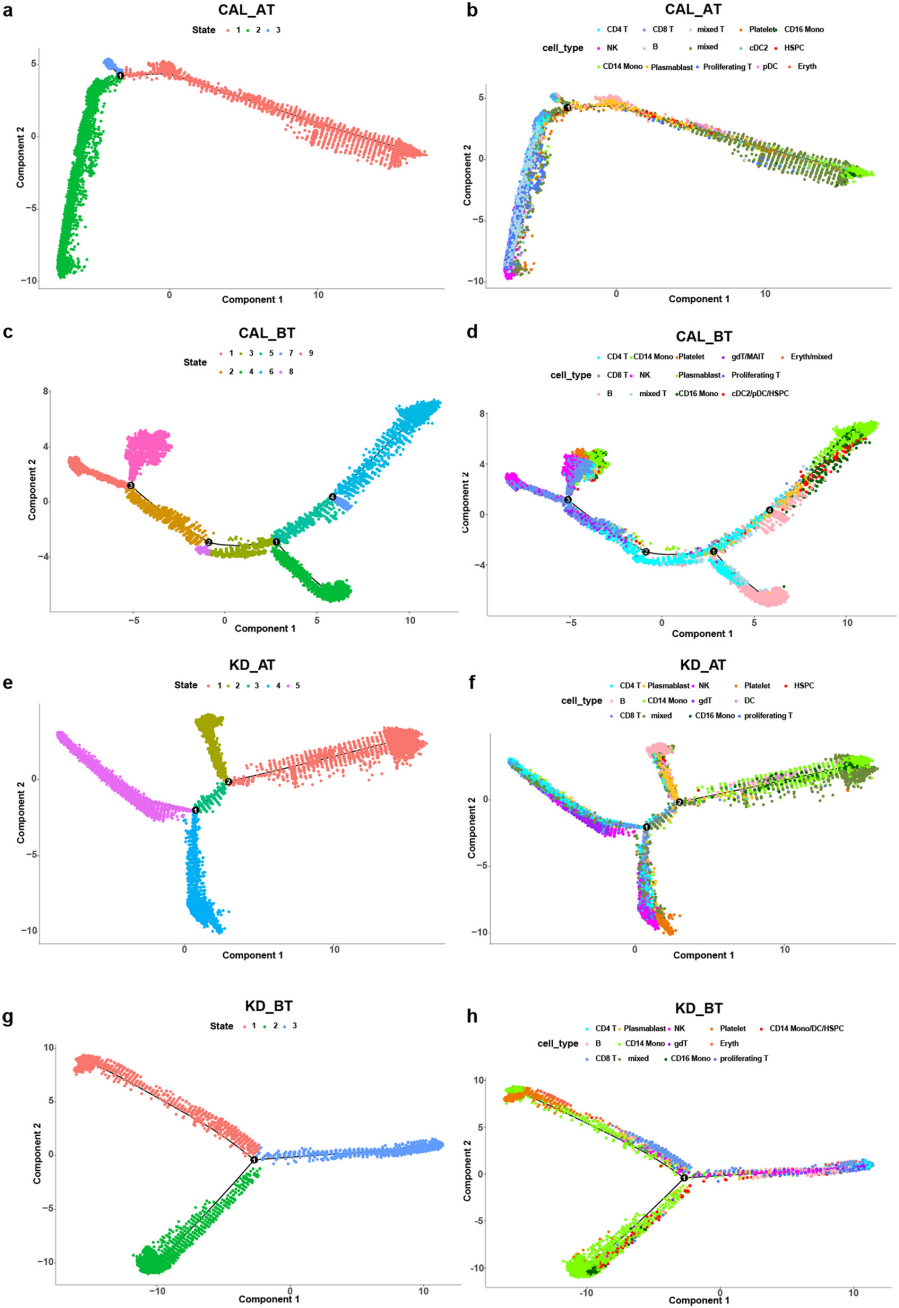
Pseudo-time analysis of cell developmental trajectory in CAL and KD patients. **(a)** The differentiation trajectory of all cells in CAL AT patients by states. **(b)** The differentiation trajectory of all cells in CAL AT patients by cell types. **(c)** The differentiation trajectory of all cells in CAL BT patients by states. **(d)** The differentiation trajectory of all cells in CAL BT patients by cell types. **(e)** The differentiation trajectory of all cells in KD AT patients by states. **(f)** The differentiation trajectory of all cells in KD AT patients by cell types. **(g)** The differentiation trajectory of all cells in KD BT patients by states. **(h)** The differentiation trajectory of all cells in KD BT patients by cell types.

Overall expression analysis shows that the genes related to adaptive immunity are highly expressed while the genes related to innate immunity are commonly repressed in CAL patients. It suggests that CAL patients may have abnormal immune cell development trajectory. The B cell development dysregulation has been studied in non-CAL KD patient (24). Whether such phenomenon is present in CAL patients is worth investigation. We used pseudo-time analysis to reconstruct the cell developmental trajectory of PBMCs in CAL AT, CAL BT, KD AT and KD BT patients. The pseudo-time analyses show that the PBMCs in CAL AT patients have three states which can be roughly classified as T/NK lineage (state 2), monocyte/B linage (state 1), and a shortened transitional state 3 (Figures 3a,b); the PBMCs in CAL BT patients have nine states which can be roughly classified as T/NK lineage (state 1 and 2), B/T lineage (state 4) and monocyte lineage (state 7), but the problem cell developmental states of CAL BT patients is that different cell types are always mixed together, e.g., B cells and T cells are mixed in state 4 while monocytes and T cells are mixed in state 1 (Figures 3c,d); the PBMCs in KD AT patients have five states which can be roughly classified as B lineage (state 2), monocyte lineage (state 1), NK/platelet linage (state 4), T lineage (state 5), and a transitional state 3 (Figures 3e,f); the PBMCs in KD BT patients have three states which can be roughly classified as monocyte/platelet lineage (state 1), T/B lineage (state 3), and monocyte lineage (state 2) (Figures 3g,h). These results demonstrates that KD AT patients have a clearly differentiated cell developmental trajectory compared to the other three groups. For CAL AT patients, B cells and monocytes failed to develop into two lineages. For CAL BT patients, not only B cells and monocytes failed to develop into two lineages, but also T cells are mixed with B cells and monocytes. For KD BT patients, B cells and T cells failed to develop into two lineages. Thus, CAL BT patients have the most disordered cell development trajectory and the poor differentiation problem for CAL AT patients is likely to be the developmental node between B cells and monocytes.

**Figure 4:**
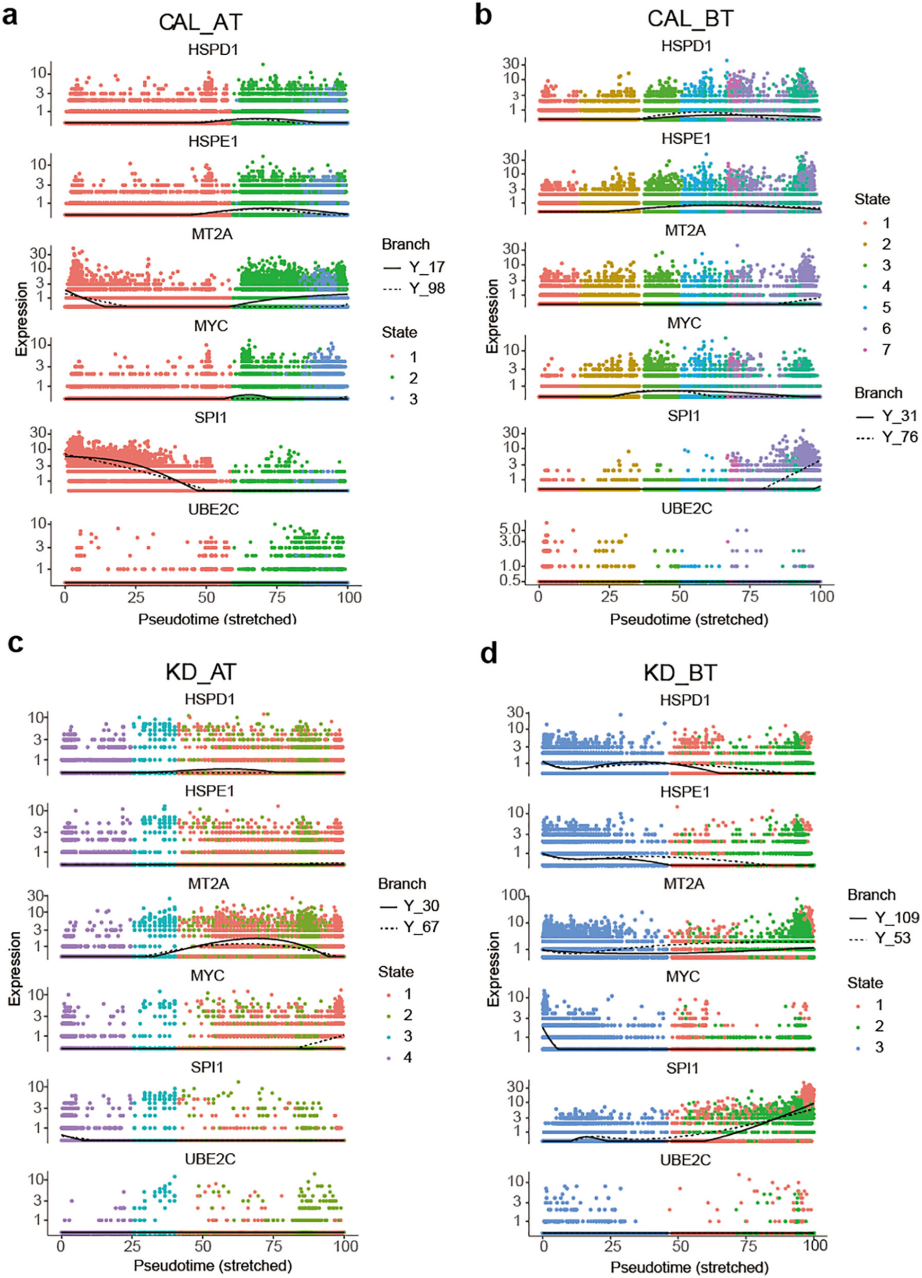
Pseudo-time analyses of expression dynamics of six B cell development and cell-cycle related genes in CAL and KD patients. **(a)** Expression dynamics of six B cell development and cell-cycle related genes in CAL AT patients. **(b)** Expression dynamics of six B cell development and cell-cycle related genes in CA BT patients. **(c)** Expression dynamics of six B cell development and cell-cycle related genes in KD AT patients. **(d)** Expression dynamics of six B cell development and cell-cycle related genes in KD BT patients.

We further examined the expression dynamic of the genes related to cell cycle and B cell development in pseudo-time analyses. The expression dynamics of UBE2C, HSPD1, HSPE1, MT2A, MYC, and SPI1 were plotted along cell developmental trajectory based on pseudo-time analysis. UBE2C, HSPD1, HSPE1, MT2A, and MYC are the genes involved in cell cycle and SPI1 activates gene expression during B cell development (24).We used HSPCs as the root for the cell developmental trajectory of each dataset, because they are the starting point of PBMCs. We found that there all six genes demonstrate a similar expression dynamic between CAL AT and KD AT patients while they exhibit a different expression dynamic among CAL BT and KD BT patients (Figures 4a–d). Both CAL AT and KD AT patients received IVIG treatment which repressed the early expression of HSPD1, HSPE1, and MYC seen in KD BT patients and restored the early expression of SPI1. The main expression dynamic differences between CAL AT and KD AT patients are that SPI1 has a much higher expression slope in the early stage of cell development and MT2A has a much earlier expression peak in CAL AT patients (Figures 4a,c). All six genes show a repression pattern in CAL BT patients and late SPI1 expression is observed in both CAL BT and KD BT patients (Figures 4b,d).

### 2.6 Cell communication patterns in CAL and KD patients

**Figure 5:**
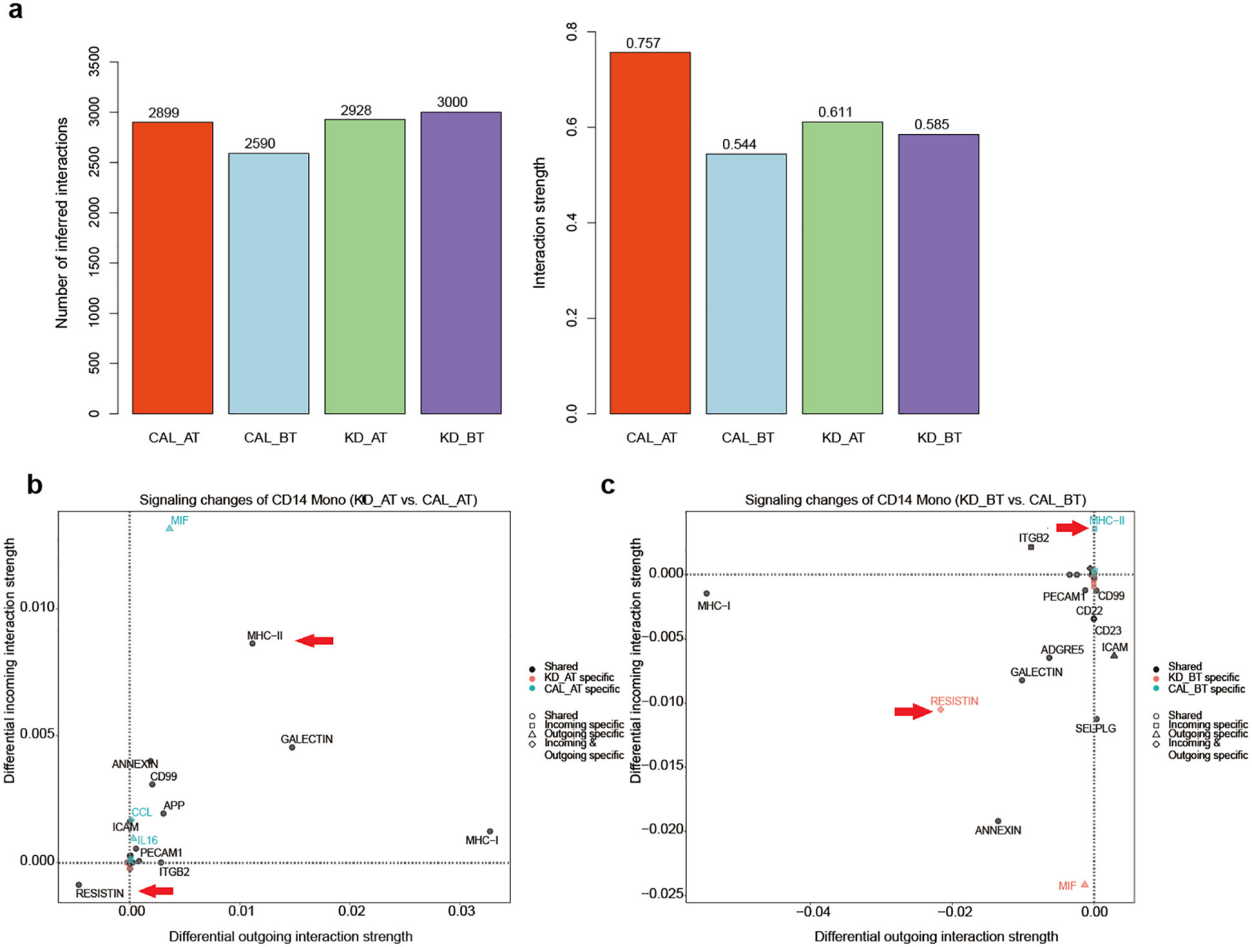
Cell-to-cell communication results in CAL and KD patients. **(a)** The number of cell-cell interactions and interaction strength in CAL AT, CAL BT, KD AT, and KD BT patients. **(b)** Signaling changes of CD14 monocytes in CAL AT patients compared to those in KD AT patients. **(c)** Signaling changes of CD14 monocytes in CAL BT patients compared to those in KD BT patients.

We first calculated the total cell-to-cell interaction numbers and strength in CAL AT, CAL BT, KD AT, and KD BT patients. They provided an overview for cell communication setting for each sample group. Among four sample groups, the PBMCs of CAL AT patients have the intermediate number of interactions but the highest interaction strength; the PBMCs of CAL BT patients have the lowest number of interactions and the weakest interaction strength; the PBMCs of KD AT patients have the second lowest number of interaction and the intermediate interaction strength while those of KD BT patients have the highest number of interactions but the second lowest interaction strength (Figure 5A). If viewing the signaling pattern in KD AT patients as normal control, it can be seen that the intermediate number of interactions with the intermediate interaction strength is a recuperative pattern for KD patients. Thus, the problem with CAL BT patients is that they have a disordered signaling pattern with both low interactions of weak strength, while the problem with CAL AT patients is that they must have some strong but bad signals that are related to CAL.

We further examined the signaling pattern among different types of cells in four patient groups. In CAL AT patients, cDC2 cells have the highest number of outgoing signals and CD16 monocytes have the highest number of incoming signals (Supplementary Figure S2a); CD4T and CD8T cells have the highest outgoing and incoming signal strength as in KD AT patients (Supplementary Figure S2b). CAL BT and KD AT patients have a similar signaling pattern among different types of cells. In both groups, cDC2 cells have the highest numbers of incoming and outgoing signals among all cell types (Supplementary Figures 2c,e); CD4T and CD8T cells have the highest outgoing and incoming signal strength among all cell types, respectively (Supplementary Figures 2d,f). In KD BT patients, CD16 monocytes have the highest number of outgoing signals and cDC2 cells have the highest number of incoming signals (Supplementary Figure 2h); CD8T cells have the highest incoming signal strength and CD14 monocytes have the highest outgoing signal strength (Supplementary Figure 2g). In both CAL AT and KD BT patients, CD14 monocytes have the most significant change of outgoing signal strength compared to those in KD AT patients. Because KD AT patients have the highest interaction strength among three sample groups, it is inferred that the signal molecules in their CD14 monocytes might contribute to CAL development.

### 2.7 Bad and good signaling molecules from CD14 monocytes

**Figure 6:**
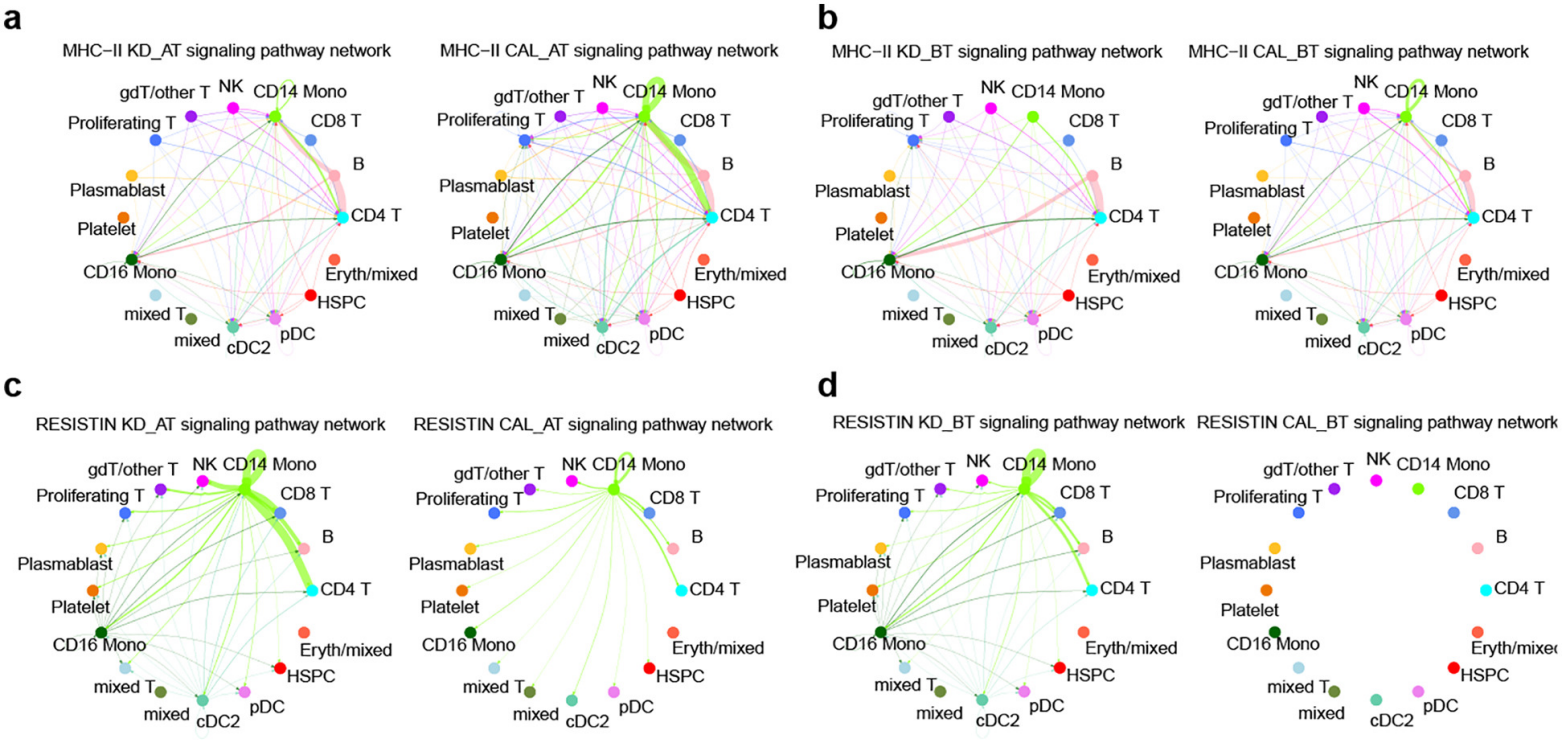
The signaling pathway network of two molecules in CAL and KD patients. **(a)** The MHC-II signaling pathway network of all cell types in CAL AT and KD AT patients. **(b)** The MHC-II signaling pathway network of all cell types in CAL BT and KD BT patients. **(c)** The RETN signaling pathway network of all cell types in CAL AT and KD AT patients. **(d)** The RETN signaling pathway network of all cell types in CAL BT and KD BT patients.

In order to study the significant signaling difference between CAL and KD groups, we compared the signaling changes of the monocytes of CAL AT to KD AT patients and CAL BT to KD patients (Figures 5b,c). Two signaling molecules from monocytes have distinctively contrasting expression patterns between CAL and KD patients. The expression of MCH-II signal molecule is significantly increased in the CD14 monocytes of CAL patients while the expression of RESISTIN is significantly increased in those KD patients (Figures 6a–d). These results propose that MCH-II is bad indicator for KD patients for its relation with CAL while RESISTIN is a good one for KD patient.

The expression profile of CD14 monocytes in CAL patients were also examined by comparing to that of KD patients. Compared CAL AT to KD AT patients, there are 19 upregulated genes in CAL AT patients (Supplementary Figure 3a). Compared CAL BT to KD BT patients, there are 131 upregulated genes in CAL BT patients (Supplementary Figure 3b). GO analyses of upregulated genes in CAL groups show that they are related to antigen processing and presentation by MHC II signaling pathway and MHC class II protein complex in both CAT AT vs. KD AT and CAL BT vs. KD BT comparisons (Supplementary Figures 3c,d). Antigen processing and presentation by MHC II signaling pathway is an important adaptive immune response. It explains why the overall expression features of CAL patients’ PBMCs exhibit an elevated level of adaptive immunity whose signaling source may be CD14 monocytes.

## 3 Discussion

KD was first reported in 1967 and its etiology remains unclear. Our previous research shows that B cell developmental dysfunction is one of cellular contributions for KD development (24). The over expression of HSPD1 and HSPE1 genes and the under expression of MYC, SPI1, MT2A and UBE2C genes are observed in the early cell development stage of KD patients before treatment. In this study, we mainly focused on the cellular features of CAL patients. After IVIG treatment, the expression of SPI1 and MT2A genes was rescued in CAL AT patients. The similar outcome could be seen in KD AT patients as well. However, the expression pattern of SPI1 and MT2A in CAL AT patients are still different from that of KD AT patients. Their early high expression explains the poor differentiation between B cell and monocytes in CAL AT patients. It further leads to the abnormal immune responses of CD14 monocytes in CAL patients.

We used scRNA-Seq to dissect the complex immune responses of PBMCs in KD and CAL. We observed that KD AT patients had an increased percentage of CD8T cell, which was also well established by previous study (24, 25). However, we did not find obvious changes in B cells and plasma cells in KD AT patients and KD BT patients, which may due to the limited samples. Further we observed no significant change of major cell types between CAL AT patients and CAL BT patients. Zheng et al. observed impaired CD8+ T cell activation and maturation in CAL patients, suggesting CD8+ T cell dysfunction during the acute phase of CAL. Chen et al. found an increasing trend in inflammatory cells (megakaryocytes and monocytes) and a decreasing trend in lymphocytes (e.g., CD4+ T, CD8+ T, mucosal-associated invariant T, natural killer, and γδ T cells) in CAL BT patients (26, 27). These findings bring forward the possible change of immune cell dynamics behind inflammatory cytokine storms and lymphopenia in the development of CAL.

Among immune cells that are involved in the pathogenesis of KD, monocytes represent 10% of leukocytes in human blood and participate in vascular inflammation including vasculitis and atherosclerosis (28). Studies suggest the activation of monocytes play an important part in the development of vasculitis during acute KD (29, 30). Furthermore, KD patients with CALs were found to have increased counts of CD14+ monocytes compared to those without CALs. The absolute counts of CD14+ monocytes form an important parameter to determine the severity of vascular damage during acute KD (31). scRNA-Seq has been used to dissect the etiology of KD and CALs since it emerged. Wang et al. found that the total production of the cytokines was expected to be elevated in acute KD patients with the increased abundance of monocytes (25). Geng et al. suggest that expanded monocyte population in KD are poorly differentiated SELL+CD14+ CD16−monocytes (32). Another scRNA-Seq study demonstrated that CD14+ monocytes mediate the inflammation in KD through the expression of FPR2 (33). Chen et al. used scRNA-Seq to study the transcriptional alterations of KD with and without CALs before IVIG treatment, and they suggested monocytes were the major source of cytokine storms, especially in patients with CALs (26). They demonstrate that IVIG combined with methylprednisolone downregulates more monocyte-driven inflammatory pathways than IVIG alone (34). Our cell-to-cell communication analyses further show that CD14 monocytes in CAL AT patients have the most significant change of outgoing signal strength compared to those in KD AT patients. Our study exhibits the importance of CD14 monocytes in CAL pathogenesis, which would support the use of lVlG combined with methylprednisolone for these patients.

Further analysis of cell-to-cell communication patterns show that the expression of MCH-II signal molecule is significantly increased in the CD14 monocytes of CAL patients, since the overall cell expression features show that they have an elevated level of adaptive immunity. Several MHC-II molecules including HLA-DRB5, HLA-DRB1, and HLA-DRA are upregulated in the CD14 monocytes of KD AT CAL patients and they all bind to CD4 receptor. It proposes that the CD14 monocyte of CAL AT patients interact with their CD4T cells and further amplify adaptive immune cascade by manipulating T cell receptor signaling pathway.

Elevated RETN expression serves as an inverse biomarker for coronary artery lesions (CALs) in Kawasaki disease (KD) patients. Resistin, a 12.5 kDa cysteine-rich secretory protein comprising 108 amino acids in humans, belongs to the resistin-like molecule (RELM) hormone family. While traditionally recognized as a pro-inflammatory cytokine expressed in immune cells that regulates chronic inflammatory, metabolic, and infectious diseases, emerging evidence reveals resistin's dual role as an innate immunity host defense peptide. This multifunctional protein demonstrates broad-spectrum antimicrobial activity, immunomodulatory capacity, and the ability to mitigate microbial product-induced inflammation (35). Nieto et al. demonstrated that resistin directly suppresses the inflammatory phenotype of monocytes and TNF-α secretion, thereby indirectly attenuating neutrophil oxidative burst (36). Our study indicates that resistin serves as a protective biomarker against coronary artery lesion (CAL) development, potentially mediated by its ability to suppress monocyte inflammatory phenotype and TNF-α production.

This study has a limited number of CAL samples which might not provide a comprehensive explanation for the development of CALs in KD patients. The samples of KD with CALs were collected after IVIG treatment. There is only a small proportion of KD patients who would develop CALs and we are unable to tell which patients would develop CALs before IVIG treatment. Therefore, obtaining samples of KD combined with CALs before IVIG treatment is difficult. We have to use public data instead. It should be mentioned that the CAL samples are younger than the non-CAL samples (average 1.5 years old vs. average 4.4 years old) in our study, which might produce variations in the clinical characteristics between two sample groups. Here we propose to establish a CAL cohort from multiple centers in future research in order to collect KD CAL patients before and after IVIG treatment, which would provide a better understanding for the pathogenesis of CALs in KD patients.

In conclusion, we delineated the single-cell atlas of PBMCs for KD patients with and without CALs in this study. KD patients with coronary artery lesions exhibit a tendency of stronger adaptive immune response and weaker innate immune response than non-CAL ones. Cell communication analyses propose significant signaling changes in the monocytes of KD patients with CAL and MCH-II is a bad signal for indicating CAL development while RESISTIN is a good signal for protecting from CAL development.

## 4 Materials and methods

### 4.1 Participants

All participants were recruited from Shanghai Children's Hospital. All manipulations were approved by the Ethics Committee of Shanghai Children's Hospital (IRB number: 2022R121). Guardians of the participants had provided their informed consent in the study. The diagnosis of complete and incomplete KD is according to 2017 American Heart Association (AHA) guidelines (1). The diagnosis of complete KD includes fever lasting for ≥5 days and the presence of ≥4 of the five principal clinical features: extremity changes, rash, conjunctivitis, oral changes and cervical lymphadenopathy. The diagnosis of incomplete KD includes fever ≥5 days, fewer than 4 of the principal clinical findings, and compatible laboratory or echocardiographic findings. The maximum internal diameter of the coronary arteries was obtained through echocardiography. These measurements were converted to *Z* scores using a model derived from the Kabayashi method (37). The CALs were defined according to the 2017 AHA Guidelines (1). Blood samples for scRNA-Seq were collected from six KD patients. The blood samples from KD group were collected on the 5th day after the onset of fever before IVIG treatment and 24 h after IVIG treatment. The blood samples of CAL AT group were collected 24 h after IVIG therapy. The diagnosis of CAL was through echocardiography inspection after IVIG therapy.

### 4.2 Public data of KD patients with CALs before IVIG treatment

We retrieved the scRNA-Seq data of KD patients with CALs before IVIG treatment from the Gene Expression Omnibus (GEO) database (accession number: GSE254657). Unfortunately, there is no detailed clinical information available for GSE254657.

#### 4.1.1 Single-cell RNA sequencing and data analyses

##### 4.1.1.1 scRNA-Seq library construction

Peripheral blood samples (2 ml each sample) were collected from the participants. PBMCs of participants were isolated according to standard density gradient centrifugation methods by using the Ficoll-Paque medium. The cell viability should exceed 90%. The single-cell library was constructed using the 5′ Library Kits. The cell suspension was loaded onto a chromium single-cell controller (10× Genomics) to generate single-cell gel beads in the emulsion (GEMs) according to the manufacturer's protocol. Lysis and barcoded reverse transcription of polyadenylated mRNA from single cells was performed inside each GEM. Post-RT-GEMs were cleaned up, and cDNA was amplified. The barcoded sequencing libraries were generated using the Chromium Next GEM Single Cell V(D)J Reagent Kits v1.1 (10× Genomics) and were sequenced as 2 × 150-bp paired-end reads on an Illumina NovaSeq platform.

##### 4.1.1.2 scRNA-Seq data processing

Cell Ranger (Version6.0.0) software was used to process the raw FASTQ files, align the sequencing reads to the GRCh38 reference transcriptome and generate a filtered UMI expression profile for each cell. Raw gene expression matrices were read into R (Version 4.2.1) and converted to Seurat objects. The number of genes, UMI counts and percentage of mitochondrial genes were examined to identify outliers. The following criteria was applied for quality control: total UMI count between 2,000 and 60,000, and mitochondrial gene percentage <5%. After removal of low-quality cells, the count matrix was normalized by SCTransform method, which is based on a negative binomial regression model with regularized parameters. Then all datasets from the individual samples were integrated using the “Find Integration Anchors” and “Integrate Data” functions in Seurat (Version 4.1.1) (38). We identified “anchors” among all individual datasets with multiple canonical correlation analysis (CCA) and used these “anchors” to create a batch-corrected expression matrix of all cells, which allowed the cells from different datasets to be integrated and analyzed. The supervised principal component analysis (SPCA) was performed to reduction and the weighted nearest neighbor (wnn) graph-based clustering was used to identify cell clusters. The cell identities were determined with multimodal reference mapping in Seurat (Version 4.1.1) (38).

To utilize scRNA-Seq data as pseudo-bulk RNA-seq data, the average gene expression value for all cells within one sample was calculated with “Average Expression” function in Seurat. Twelve scRNA-Seq average expression results was integrated together and then used as pseudo-bulk RNA-seq data for PCA analysis.

##### 4.1.1.3 Differential expression and functional enrichment analysis

DEG analysis for each cell type on the sample level following the recommendation of Bioconductor (39). The differential expression analysis was conducted between conditions by using Libra (Version 1.0.0) (40), which implements a total of 22 unique differential expression methods that can all be accessed from one function. We used “run_de” functions with pseudo-bulk approach, implementing the DESeq2 (Version 1.36.0) (41) with a likelihood ratio test. GSEA pathway analysis was performed using cluster Profiler (Version 4.7.1) (32, 42). Hallmark gene sets in the Molecular Signatures Database (MSigDB Version7.1) (43) served as the gene function database. *P*-values were adjusted to FDRs. FDRs <0.05 was chosen as the cut-off criterion indicating a statistically significant difference.

##### 4.1.1.4 Pseudo-time analysis of cell differentiation trajectories

Pseudo-time analysis of cell differentiation trajectories for each sample dataset was performed with R package Monocle 2 (44).The expression feature and inferred cell type for each sample dataset from Seurat result was used to construct the cell dataset for Monocle analysis pipeline. We used the Monocle built-in approach named “dpFeature” to detect the variable genes that define cell's differentiation. Its advantages are needing no prior biological knowledge and discovering important ordering genes from data itself. Dimension reduction was performed with 2 max components and “DDR Tree” method. HSPCs and B cells in each sample dataset for pseudo-time analysis were extracted according to the cell identities from Seurat result.

##### 4.1.1.5 Cell-cell communication analysis

Cell-cell communication analysis was performed with R package CellChat 1.6.1 (45). Human database in CellChat was set to be the default ligand-receptor pair for cell-to-cell communication analysis.

## Data Availability

The original contributions presented in the study are included in the article/Supplementary Material, further inquiries can be directed to the corresponding authors.

## Supplementary material

Supplementary Figure S1 Integrated single-cell profiling of PBMCs in CAL and KD patients. **(A)** Integrated single-cell profiling of PBMCs in CAL and KD patients. **(B)** The inferred cell types marked with different colors based on multimodal reference mapping. **(C)** Expression of canonical gene markers for each cell type in CAL and KD patients based on integration analysis.

Supplementary Figure S2 Cell-to-cell communication patterns in CAL and KD patients. **(a)** The incoming and outgoing interaction numbers of all cell types in CAL AT patients. **(b)** The incoming and outgoing interaction strength of all cell types in CAL AT patients. **(c)** The incoming and outgoing interaction numbers of all cell types in CAL BT patients. **(d)** The incoming and outgoing interaction strength of all cell types in CAL BT patients. **(e)** The incoming and outgoing interaction numbers of all cell types in KD AT patients. **(f)** The incoming and outgoing interaction strength of all cell types in KD AT patients. **(g)** The incoming and outgoing interaction numbers of all cell types in KD BT patients. **(h)** The incoming and outgoing interaction strength of all cell types in KD BT patients.

Supplementary Figure S3 Expression analyses of CD14 monocytes in CAL and KD patients. **(a)** Volcano plot of upregulated and downregulated genes in the CD14 monocytes of CAL AT patients compared to those of KD AT patients. **(b)** Volcano plot of upregulated and downregulated genes in the CD14 monocytes of CAL BT patients compared to those of KD BT patients. **(c)** GO term enrichment analysis of 19 upregulated genes in the CD14 monocytes of CAL AT patients compared to KD AT patients. **(d)** GO term enrichment analysis of 131 upregulated genes in the CD14 monocytes of CAL BT patients compared to KD BT patients.
