# Supplementary figures and images for: Delineation of single-cell Altas provides new insights for development of coronary artery lesions in Kawasaki disease: bad and good signaling molecules
Source: Front Pediatr. 2025 Jun 25;13:1596643. doi: 10.3389/fped.2025.1596643 (PMC12237900; doi:10.3389/fped.2025.1596643)

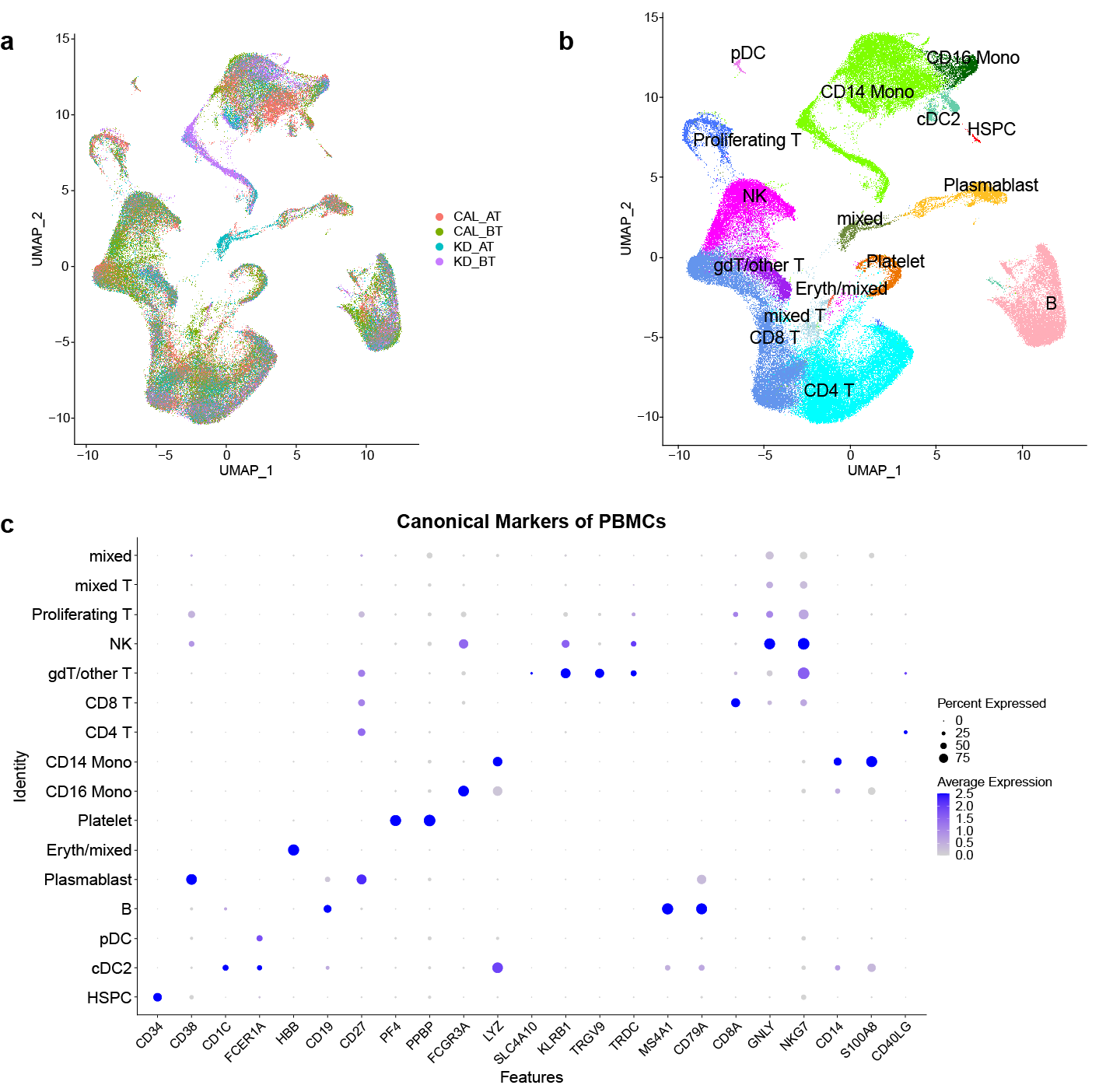

Supplement: Supplementary Figure S1 — Integrated single-cell profiling of PBMCs in CAL and KD patients. (A) Integrated single-cell profiling of PBMCs in CAL and KD patients. (B) The inferred cell types marked with different colors based on multimodal reference mapping. (C) Expression of canonical gene markers for each cell type in CAL and KD patients based on integration analysis. [file Image1.tif]

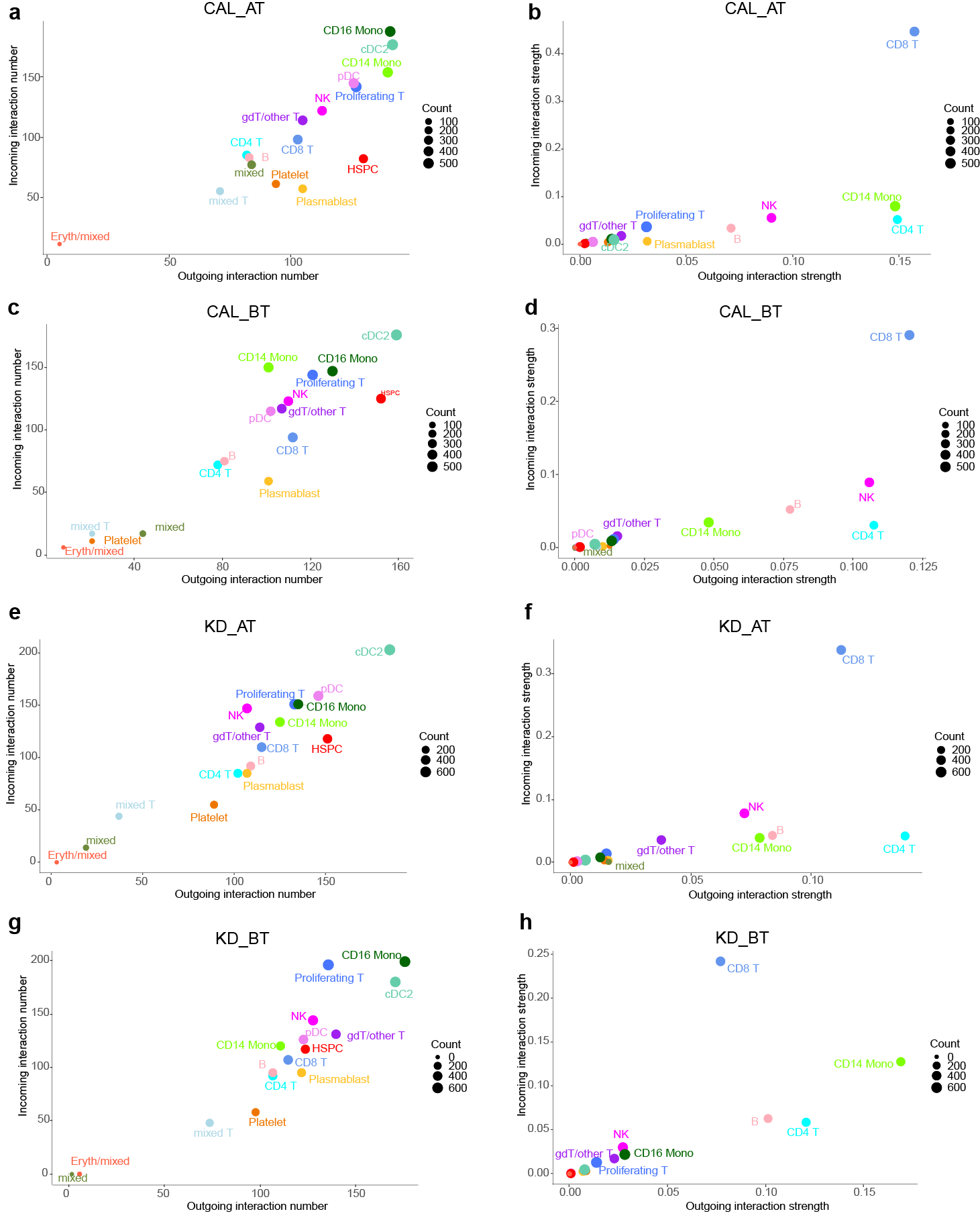

Supplement: Supplementary Figure S2 — Cell-to-cell communication patterns in CAL and KD patients. (a) The incoming and outgoing interaction numbers of all cell types in CAL AT patients. (b) The incoming and outgoing interaction strength of all cell types in CAL AT patients. (c) The incoming and outgoing interaction numbers of all cell types in CAL BT patients. (d) The incoming and outgoing interaction strength of all cell types in CAL BT patients. (e) The incoming and outgoing interaction numbers of all cell types in KD AT patients. (f) The incoming and outgoing interaction strength of all cell types in KD AT patients. (g) The incoming and outgoing interaction numbers of all cell types in KD BT patients. (h) The incoming and outgoing interaction strength of all cell types in KD BT patients. [file Image2.tif]

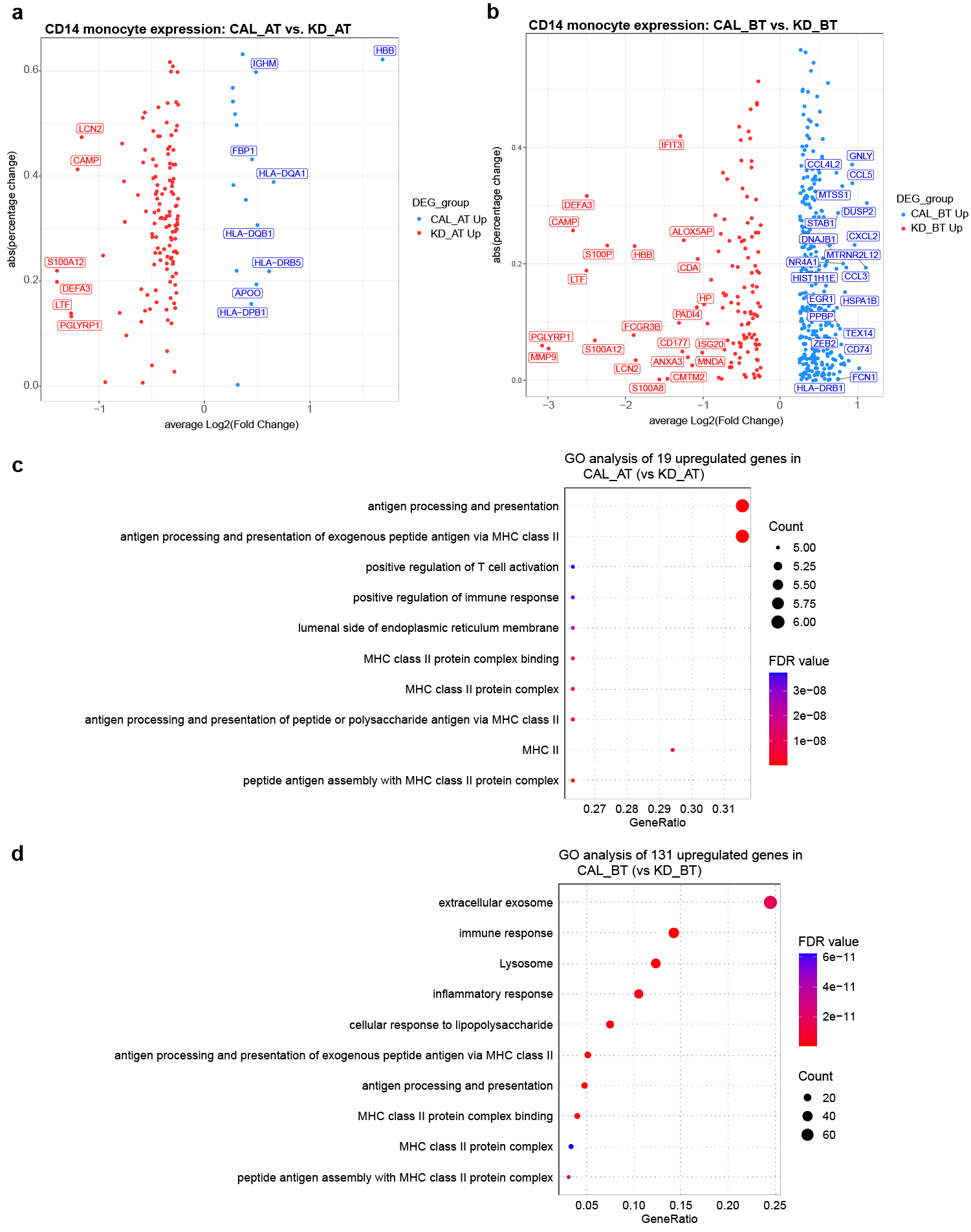

Supplement: Supplementary Figure S3 — Expression analyses of CD14 monocytes in CAL and KD patients. (a) Volcano plot of upregulated and downregulated genes in the CD14 monocytes of CAL AT patients compared to those of KD AT patients. (b) Volcano plot of upregulated and downregulated genes in the CD14 monocytes of CAL BT patients compared to those of KD BT patients. (c) GO term enrichment analysis of 19 upregulated genes in the CD14 monocytes of CAL AT patients compared to KD AT patients. (d) GO term enrichment analysis of 131 upregulated genes in the CD14 monocytes of CAL BT patients compared to KD BT patients. [file Image3.tif]
